# Listening to Music Through Hearing Aids: Potential Lessons for Cochlear Implants

**DOI:** 10.1177/23312165211072969

**Published:** 2022-02-18

**Authors:** Brian C. J. Moore

**Affiliations:** 1 Cambridge Hearing Group, Department of Psychology, 2152 University of Cambridge, Cambridge, England

**Keywords:** music, dynamic range, automatic gain control, hearing aids, cochlear implants, compression speed

## Abstract

Some of the problems experienced by users of hearing aids (HAs) when listening to music are relevant to cochlear implants (CIs). One problem is related to the high peak levels (up to 120 dB SPL) that occur in live music. Some HAs and CIs overload at such levels, because of the limited dynamic range of the microphones and analogue-to-digital converters (ADCs), leading to perceived distortion. Potential solutions are to use 24-bit ADCs or to include an adjustable gain between the microphones and the ADCs. A related problem is how to squeeze the wide dynamic range of music into the limited dynamic range of the user, which can be only 6–20 dB for CI users. In HAs, this is usually done via multi-channel amplitude compression (automatic gain control, AGC). In CIs, a single-channel front-end AGC is applied to the broadband input signal or a control signal derived from a running average of the broadband signal level is used to control the mapping of the channel envelope magnitude to an electrical signal. This introduces several problems: (1) an intense narrowband signal (e.g. a strong bass sound) reduces the level for *all* frequency components, making some parts of the music harder to hear; (2) the AGC introduces cross-modulation effects that can make a steady sound (e.g. sustained strings or a sung note) appear to fluctuate in level. Potential solutions are to use several frequency channels to create slowly varying gain-control signals and to use slow-acting (or dual time-constant) AGC rather than fast-acting AGC.

## Introduction

Users of both hearing aids (HA) and cochlear implants (CI) experience problems when listening to music (Chasin & Hockley, 2014; Looi et al., 2008; Madsen & Moore, 2014; McDermott, 2004; Moore, 2016). Some of these problems are related to the reduced resolution and processing capacity of the impaired auditory system (Moore, 2003; Moore, 2007; Shannon, 1983). For example, both HA users and CI users have reduced spectral resolution (Friesen et al., 2001; Glasberg & Moore, 1986; Pick et al., 1977). For CI users, this reduction is very substantial because of the spread of current within the cochlea, and so it is likely to severely limit the ability to “hear out” one instrument or voice in the presence of another instrument or voice (Mehta & Oxenham, 2017). This in turn will limit the enjoyment of any music that includes multiple instruments and voices (Limb & Roy, 2014) and this limitation is unlikely to be alleviated by changes in CI processing, unless a way can be found of making the electrical stimulation much more place selective. However, other problems are related to the design of the HAs and CIs and these problems can potentially be reduced by improvements in design. This paper considers some of the lessons that have been learned from studies of music perception via HAs and describes how those lessons might be applied to improving the design of CIs.

In both HAs and CIs, the broadband signals picked up by the microphone(s) are passed through an array of bandpass filters to create channel signals corresponding to the outputs of filters with different centre frequencies. In HAs, the channel signals are processed in various ways before being combined to create a broadband signal that is delivered to a miniature loudspeaker (called a receiver) that generates the output sound. In CIs the channel signals are used to create electrical signals that are delivered to the individual electrodes in the array implanted within the cochlea. Hence, in CIs, the number of channels is usually equal to the number of implanted electrodes, although the effective number of independent channels is smaller than the number of electrodes, because of current spread in the cochlea (Friesen et al., 2001). In a CI, the signal in the channel with the lowest centre frequency is usually used to derive the electrical signal delivered to the most apical electrode in the cochlea, while the signal in the channel with the highest centre frequency is usually used to drive the most basal electrode, with a continuous gradation in between. This represents an attempt to recreate the tonotopic mapping of frequency to place that occurs within a normal cochlea (von Békésy, 1960). Thus, information about the short-term spectrum of sounds is conveyed by the relative strength of the electrical signals across the electrode array.

## Requirements for Music Listening With CIs

It is helpful first to consider the properties that a CI should have in order to improve the experience of listening to music. The key properties are:

1. Envelope cues in different frequency regions, which are the main source of auditory information provided by CIs (Clark et al., 1990; Wilson et al., 1991; Zeng et al., 2003), should be preserved and coded as faithfully as possible.
2. Nonlinear distortion occurring prior to filtering of the signal into frequency bands or channels should be minimal. This is because nonlinear distortion leads both to changes in the shape of the waveform and to frequency components in the short-term spectrum that are not present in the original signal, giving a misleading representation of the spectral shape of a sound (Tan et al., 2003). Nonlinear distortion introduced after the signal is filtered into frequency channels in a CI does not have the same effect on the representation of the spectrum of a sound, but it can distort the representation of the envelope in each channel.
3. The wide range of sound levels that occur in music, especially live music, must be compressed into the narrow range of electrical current values between the detection threshold and the highest comfortable level. This must be done while preserving the representation of the envelope of the sound in each frequency channel as well as possible.

As is discussed below, these properties are not easy to achieve, and the current state of the art is far from optimal in this respect.

## Sources of Envelope Distortion

In this section, I consider some (but not all) of the sources of envelope distortion in HAs and CIs. Envelope distortion introduced by automatic gain control (AGC) systems is discussed in a later section. Most HAs and CIs incorporate various forms of adaptive signal processing, i.e. signal processing that changes over time in response to changes in the input signal. Examples are noise reduction and adaptive directional processing. These forms of signal processing, because they are time-varying, inevitably distort the envelopes of the channel signals in HAs and CIs.

Noise-reduction systems can be applied to the signal from a single microphone. They have been designed to improve the ability to understand speech in the presence of background noise. They generally work by estimating the momentary speech-to-noise ratio in each channel and applying attenuation to the channels with the poorest estimated speech-to-noise ratio (Hamacher et al., 2006; Holube et al., 1999; Launer et al., 2016; Yang & Fu, 2005). In more recent approaches, artificial neural networks have been used to process speech in background sounds for application both to HAs (Healy et al., 2021; Keshavarzi et al., 2019) and CIs (Goehring et al., 2017; Goehring et al., 2019).

All single-microphone noise-reduction systems involve a trade-off; the more the background noise is reduced relative to the speech, the more distortion there is, including envelope distortion. There appear to be large individual differences in preferences for noise-reduction systems and in preferences for the amount of noise reduction, some people being “noise haters” and others being “distortion haters” (Brons et al., 2012). In any case, since these noise-reduction systems have been designed to improve the perception of speech in noise, they are unlikely to be of any benefit when listening to music. Rather, the envelope distortion that they introduce is likely to degrade the perception of music by users of HAs and CIs. Therefore, it is recommended that a CI is set up with a dedicated music program, as is often done for HAs, and in the music program any adaptive noise-reduction processing is disabled.

A second form of adaptive processing, directional processing, is used when two or more microphones are available, as is the case with most HAs and CIs. Some (but not all) such systems are based on the assumption that the “target” sound that the user wishes to hear comes from the front. They attempt to estimate, for each frequency channel, the direction of the most prominent interfering sound coming from the sides or back, and to create a null in the directional response so as to attenuate that interfering sound (Launer et al., 2016). This creates time-varying changes in the effective frequency response of the HA or CI for sounds coming from the sides or back, and also distorts the envelope representation of such sounds. Again, this is likely to impair music perception, since music often involves spatially distributed sound sources. Even for directional processing systems that attempt to preserve sounds of interest from several directions, the processing is adaptive and time varying, and is likely to result in the introduction of spurious amplitude modulation. As for noise-reduction processing, it is recommended that a CI is set up with a dedicated music program and that any adaptive directional processing is disabled for that program.

## The Problem of the Dynamic Range of the Input Signal

Most CIs and some HAs use 16-bit analogue-to-digital converters (ADCs) to digitise the microphone signals (Launer et al., 2016; Zakis, 2016). In theory this can code a 96-dB range of input sound levels (6 dB per bit). In practice the achieved range is typically 85–90 dB, because of microphone noise, noise in the ADCs themselves, and noise in the electronic circuitry. Although in principle the dynamic range can be increased by the application of a low-level spectrally shaped noise called dither (Vanderkooy & Lipshitz, 1984), this is not to my knowledge applied in HAs or CIs. In most HAs and CIs, the gain of the pre-amplifier between the microphone and the ADC is set so that the lowest sound level that can be coded is about 15 dB SPL, which means that the highest sound level that can be coded is 100–105 dB SPL. When listening to music in the home, peak sound levels rarely exceed 95 dB SPL and most CIs and HAs can handle this without significant distortion. However, when listening to live music, or amplified music in a club or discotheque, peak sound levels can reach 115–120 dB SPL (Chasin & Hockley, 2014; Hockley et al., 2012). Sound at such levels can be unpleasant and may appear distorted even for people with normal hearing, because of nonlinear distortion produced in the outer and middle ear (Price & Kalb, 1991) and because of the effects of upward spread of masking (Studebaker et al., 1999). However, for users of HAs and CI, such high sound levels can also lead to overload (peak clipping) that reduces the perceived sound quality, at least for users of HAs (Tan & Moore, 2008). Apart from introducing spectral changes in the signal, peak clipping results in a distortion of the envelope cues that are important for CI users.

There are several solutions to this problem. One is to use 24-bit ADCs, a solution that has been adopted by several manufacturers of HAs, even though it decreases battery life. Another solution is to include an adjustable gain between the microphones and the ADCs; this gain adjustment can be compensated for in the subsequent digital-processing stages of the HA or CI (Hockley et al., 2012; Zakis, 2016). This is done in some but not all HAs and CIs. It is recommended, therefore, that the range of signal levels that can be handled by CIs without distortion is increased, either via the use of 24-bit ADCs or via the use of an adjustable gain between the microphones and the ADCs.

## Squeezing Music Into the Limited Dynamic Range of the CI User

### The Perceptual Dynamic Range for Users of HAs and CIs

Much recorded music, and music that is broadcast or transmitted via the internet, is subjected to some form of amplitude compression to reduce its dynamic range (the difference between the highest level and lowest level in the music) (Croghan et al., 2014; Hjortkjær & Walther-Hansen, 2014). This is especially true for “pop” music. However, live music, especially classical music, can have a very wide dynamic range; the peaks occurring during a *fortississimo* (*fff*) passage may be 70 dB or more above the dips in level in a *pianississimo* (*ppp*) passage (Hockley et al., 2012; Kirchberger & Russo, 2016). The perceptual dynamic range – defined here as the range between the detection threshold and the level at which sound starts to become uncomfortably loud – is typically at least 95–100 dB for people with normal hearing (Moore, 2012). This is enough to allow the *pianississimo* passages to be heard without the *fortississimo* passages being uncomfortably loud, except perhaps when a *pianississimo* passage occurs immediately after a *fortississimo* passage. However, hearing loss usually results in loudness recruitment (a more rapid than normal growth of loudness with increasing sound level, once the sound level exceeds the elevated detection threshold) and a reduced perceptual dynamic range (Fowler, 1936; Steinberg & Gardner, 1937). For a hearing loss of, say, 70 dB, the perceptual dynamic range is typically only about 30 dB (Miskolczy-Fodor, 1960; Moore, 2004; Moore & Glasberg, 2004). For electrical pulses delivered directly to an individual electrode in a CI, the perceptual dynamic range is typically only 6–20 dB (Fourcin et al., 1979; Zeng, 2004). Hence some form of amplitude compression is essential to squeeze the wide range of sound levels encountered during performances of live music into the limited perceptual dynamic range of users of HAs and CIs.

### Amplitude Compression in HAs

In HAs, amplitude compression is usually achieved via multi-channel AGC. The digitised microphone signal is filtered into several frequency channels (typically between 3 and 24) and independent AGC is applied in each channel. A basic characteristic of an AGC system is its input-output function, a plot of the output level as a function of the input level, both in dB. For many AGC systems, the gain (output level minus input level) is independent of the input level for low input levels. Hence, the input-output function has a slope of one. This is called linear amplification. At higher input levels, the gain decreases progressively with increasing input level, and the input-output function has a slope of less than one. The compression threshold is defined as the input level at which the gain is reduced by 2 dB, relative to the gain applied in the region of linear amplification (ANSI, 2014). One reason for having a compression threshold is that it is impractical to continue to increase the gain indefinitely as the input level decreases because this would make microphone noise or low-level environmental noises intrusive. Indeed, for very low input levels, the gain in many HAs is reduced to prevent such noises from being audible; this is called expansion, and it is associated with a slope of the input-output function greater than one.

Over the range of input levels where compression is applied, the “amount” of compression is specified by the compression ratio, the change in input level (in dB) required to achieve a 1-dB change in output level (for an input level above the compression threshold). For example, a compression ratio of 3 means that each 3-dB increase in input level leads to a 1-dB increase in output level, so the input-output function has a slope of 1/3. In most HAs the compression ratio is set independently for each channel depending on the perceptual dynamic range at the centre frequency of that channel, which is often estimated from the audiometric threshold (Keidser et al., 2012; Moore et al., 2010).

AGC systems vary in how rapidly they respond to a sudden change in the input sound level. The speed of the AGC systems in HAs is typically measured using a sound whose input level changes abruptly between 55 and 90 dB SPL (ANSI, 2014). When the sound level increases, the gain decreases. The time taken for the output to get within 3 dB of its steady value is called the attack time. When the sound level decreases, the gain increases. The time taken for the output to increase to within 4 dB of its steady value is called the recovery time or release time. For HAs, there is no general consensus about what compression speed is “best” (Moore, 2008). Some manufacturers use fast-acting compression, typically with attack times in the range 0.5–20 ms and release times in the range 5–150 ms. Some manufacturers use slow-acting compression, typically with attack times in the range 20–2,000 ms and release times in the range 500–5,000 ms or more. The attack time is usually chosen to be smaller than the release time to reduce the experience of uncomfortable loudness when sudden increases in sound level occur. Some manufacturers use systems with two or more speeds (Moore et al., 1991; Moore & Glasberg, 1988; Nordqvist & Leijon, 2004; Stone et al., 1999; Stöbich et al., 1999). In these systems, the gain is mostly determined by a slow-acting AGC system, but a fast-acting system takes over and rapidly reduces the gain if there is a sudden increase in sound level. If the increase in sound level is brief (for example the sound of a door slamming or a knife being dropped on a plate), the gain returns to the value set by the slow-acting system.

There have been several studies of preferences for compression speed for HA users when listening to music. Hansen (2002) used a simulated 15-channel hearing aid and a paired-comparison procedure. The gain for medium input sound levels was adjusted for each subject using the NAL-R fitting rule (Byrne & Dillon, 1986), but the compression ratio was fixed at 2 for all channels and subjects, rather than being tailored to suit the individual hearing losses. The stimuli included classical and pop music presented at an input level of 80 dB SPL. The longest release time used (4,000 ms) was significantly preferred over the two shorter release times used (400 and 40 ms).

Moore et al. (2011) used a paired-comparison procedure and a simulated five-channel HA to compare preferences for slow versus fast compression, using both speech and music stimuli and using three mean levels for the input signal, 50, 65 and 80 dB SPL. The simulated HA was programmed to suit the individual hearing losses, which were mild, using the CAM2 procedure (Moore et al., 2010). They found slight mean preferences for slow compression for both speech and music, but the effect was only clear for an input level of 80 dB SPL, and not for input levels of 50 and 65 dB SPL. They also assessed the effect of slightly delaying the audio signal relative to the gain-control signal (by 2.55 to 15 ms). This delay, referred to as the “alignment delay”, allowed the gain to be reduced just *before* any sudden increase in sound level (Robinson & Huntington, 1973; Verschuure et al., 1993), thus increasing the effectiveness of the AGC system in preventing loudness discomfort. The effect of the alignment delay was small except for a highly percussive sound (solo xylophone) combined with the fast compression system, for which alignment delays of 2.5, 5, and 7.5 ms were preferred over no delay.

Croghan et al. (2014) used a paired-comparison procedure and a simulated HA with either 3 or 18 channels to compare preferences for slow versus fast compression. They used both rock and classical music, and the stimuli were subjected to various amounts of compression *prior to* the simulated HA processing, to simulate the compression that is often applied to recorded music. The stimuli were presented at a nominal level of 65 dB SPL prior to the simulated HA processing. The HAs were individually fitted using the NAL-NL1 prescription method (Byrne et al., 2001). For stimuli that were not compressed prior to HA processing, slow compression was preferred over fast compression for both the 3- and 18-channel systems and for both classical and rock music.

Moore and Sęk (2016b) used a paired-comparison procedure and a simulated five-channel HA to compare preferences for slow versus fast compression. Three mean levels were used for the input signal, 50, 65 and 80 dB SPL. They tested a hypothesis put forward by Moore (2008) that reduced sensitivity to the temporal fine structure (TFS) of sounds may be associated with a preference for slow compression. This hypothesis is based on the observation that high sensitivity to TFS is associated with a better ability to understand speech at low speech-to-background ratios (Füllgrabe et al., 2015), perhaps because TFS information is useful for the perceptual segregation of the target and background sounds (Lunner et al., 2012). Also, high sensitivity to TFS may be associated with less reliance on envelope cues in different frequency channels and with more resistance to the envelope distortion produced by fast-acting compression (Souza, 2002; Stone & Moore, 1992, 2004; Stone et al., 2009). On the other hand, people with poor sensitivity to TFS, including users of CIs, may rely mainly on envelope cues in different frequency channels, and for them it is important to avoid the envelope distortion that is introduced by fast compression. Hence, slow compression may be the preferred option for HA users and CI users with poor sensitivity to TFS.

Moore and Sęk (2016b) tested subjects with moderate-to-severe sensorineural hearing loss and assessed their sensitivity to TFS indirectly by measuring difference limens for frequency (DLFs) for a pure tone signal centred at 2,000 Hz, based on the (not universally accepted) assumption that frequency discrimination depends on the use of TFS information for frequencies up to a few kHz (Heinz et al., 2001a; Heinz et al., 2001b; Moore, 1973; Moore & Ernst, 2012). The simulated HA was programmed to suit the individual hearing losses using the CAM2 prescription method (Moore et al., 2010), modified slightly as described in Moore and Sęk (2013) and Moore and Sęk (2016a). Only one ear of each subject was tested.

**Figure 1.**
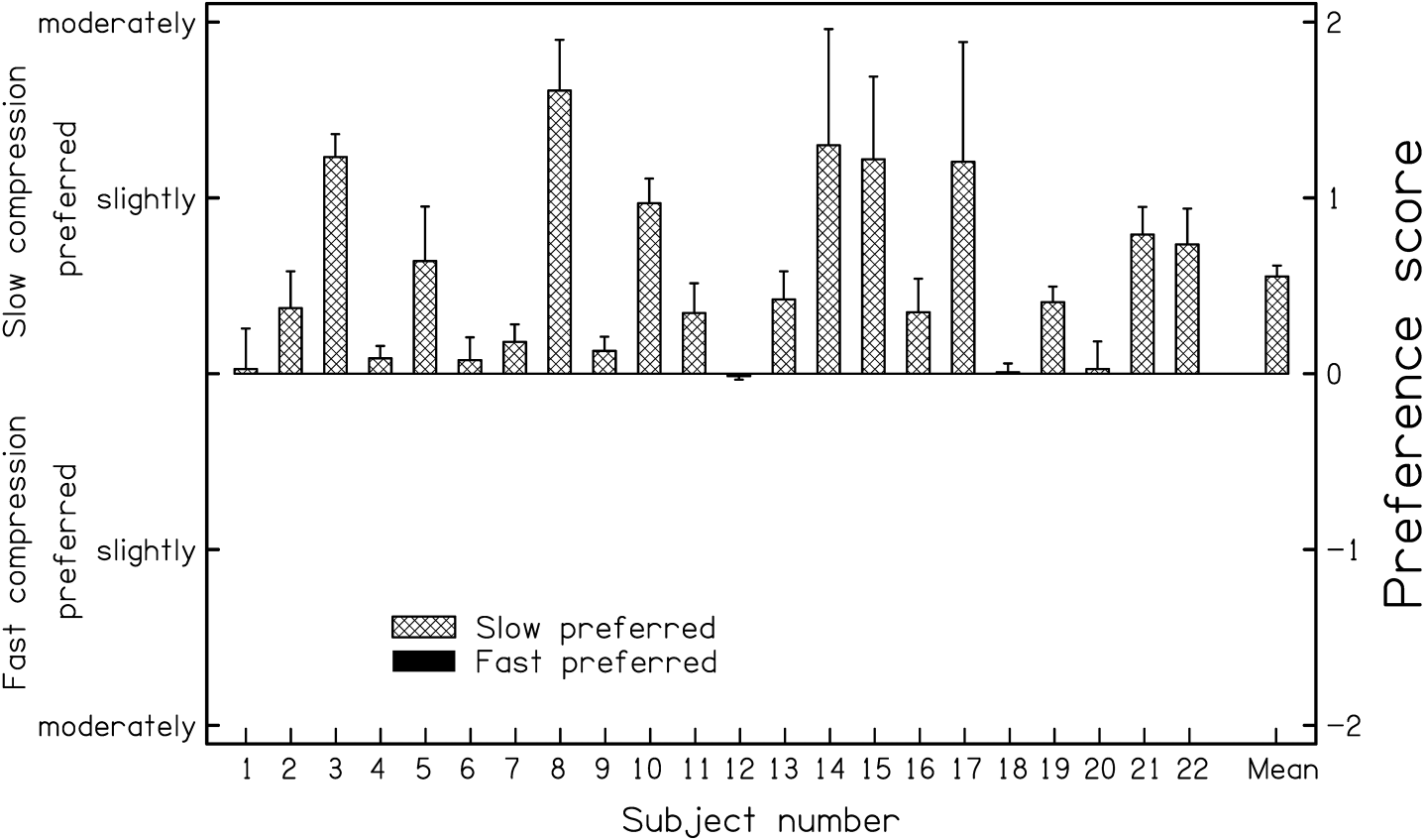
Preferences for slow versus fast compression for individual subjects and for the mean across subjects, as obtained by Moore and Sęk (2016b) using a simulated HA. Bars falling above the horizontal line at the centre of the panel indicate preferences for slow compression over fast compression. Redrawn from Moore and Sęk (2016b).

For all three input levels used, there were small overall preferences for slow over fast compression. Figure 1 shows the individual and mean preferences averaged across the three levels for listening to music. Preference scores above 0 indicate a preference for slow compression. Nine subjects showed a preference for slow compression of 0.5 scale units or more (the scale went from −3 to +3, with 0 indicating no preference), and the rest showed very small preferences for slow compression or no clear preference. Thus, at least for the music stimuli used in the study (jazz music, classical music and a man singing with a guitar), slow-acting compression seems to be a “safe” option. The logarithms of the DLFs for the test ears were weakly but significantly correlated with preference scores for music, based on a one-tailed test: *r* = 0.39, *p* < 0.05. This provides some weak support for the hypothesis that poorer sensitivity to TFS (indicated by larger DLFs) is associated with stronger preferences for slow compression.

In summary, studies of hearing-impaired subjects using simulated HAs suggest that slow compression is preferable to fast compression for listening to music. However, it should be remembered that while the studies described above used a reasonably wide range of input levels (50–80 dB SPL), the range was not as wide as might be encountered when listening to live music. Also, the stimuli did not include conditions where the level changed abruptly from a high to a low value, as might occur in music. When the level decreases abruptly, the gain in a slow-acting compression system takes some time to increase; for example, the recovery time of the slow-acting compression used by Moore and Sęk (2016b) was 3,000 ms. For users of HAs the subjective effect of an abrupt decrease in level is that the HA has become “dead” for a second or two, which is clearly undesirable.

### Amplitude Compression in CIs

**Figure 2.**
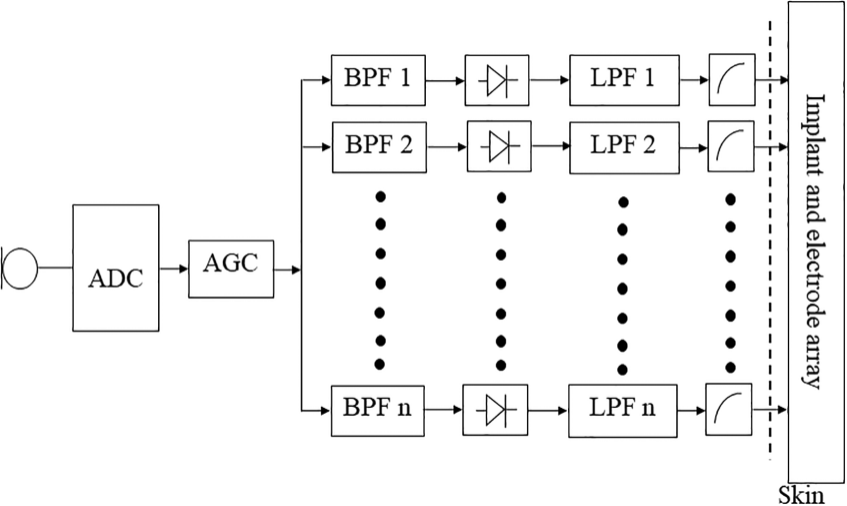
Simplified schematic diagram of the signal processing preformed in several CIs.

Because of the very small perceptual dynamic range for electrical signals applied to the auditory nerve, all CIs incorporate some form of amplitude compression. Figure 2 is a simplified schematic of the signal processing that is performed in many CIs. The analogue microphone signal is subjected to analogue-to-digital conversion (ADC) (often after pre-amplification and filtering to reduce the influence of strong low-frequency components) and the broadband digital signal is subjected to AGC. The amplitude-compressed signal is then filtered into frequency channels using an array of bandpass filters (BPF, 1 to *n*), the channel signals are rectified and lowpass filtered (LPF) to extract the envelope, the envelope signals are subjected to instantaneous compression (often referred to as mapping), and the compressed envelope is used to modulate the amplitude or width of the biphasic current pulses delivered to each electrode. Thus, there are two stages of amplitude compression, produced by the front-end AGC and by the mapping for the individual channels. The CIs produced by three of the main manufacturers, Cochlear Corporation, Med-El, and Advanced Bionics, all have this general structure, although they differ in the speed of the front-end AGC, the number of channels, the sharpness of the filters used to create the channel signals, and the method of envelope extraction (Vaerenberg et al., 2014). The early devices made by Cochlear Corporation have a front-end slow-acting “automatic sensitivity control” (Seligman & Whitford, 1995) followed by a fast-acting AGC system, with a very high compression ratio (called a compression limiter) (Khing et al., 2013). The current CIs manufactured by Med-El, Advanced Bionics, and Cochlear Corporation incorporate a dual time-constant system (Boyle et al., 2009; Khing et al., 2013; Stöbich et al., 1999) similar to that described by Stone et al. (1999). The CIs manufactured by Cochlear Corporation also include the option of a multi-channel slow-acting AGC system called adaptive dynamic range optimisation (ADRO, Blamey, 2005) which is applied separately to the envelope signal in each channel (Khing et al., 2013; Wolfe et al., 2011). To my knowledge, there are no published evaluations of the ADRO system for music listening.

When listening to music, there are several problems associated with the use of single-channel AGC applied to the broadband signal:

1. The input-output functions of the front-end AGCs used in CIs have been designed to work well for the typical speech signals encountered in everyday life but may not be optimal for music. For example, the Advanced Bionics system uses a compression threshold of about 63 dB SPL combined with a high compression ratio (about 12). This means that many music signals would be subjected to strong (albeit slow-acting) amplitude compression, reducing the impression of dynamic changes in music, such as the contrast between a *forte* passage and a *piano* passage. In comparison, the multi-channel AGC systems used in HAs typically have compression thresholds in the range 20 to 45 dB SPL and compression ratios in the range 1.5 to 3 (Moore et al., 2001).
2. A relatively intense narrowband signal (e.g. a strong bass sound) will reduce the level for *all* frequency components, making some parts of the music harder to hear.
3. The AGC introduces cross-modulation effects (Stone & Moore, 2004; Stone & Moore, 2008): a brief strong signal from one source (e.g. a drum beat) leads to a reduction in gain for another sound source (e.g. strings). This can make a steady sound (e.g. sustained strings or a sung note) appear to fluctuate in level and it may make it harder to segregate sound sources. Such effects are strongest for fast-acting compression, but they occur to some extent even when slow compression is used, unless it is very slow indeed.

Improvements might be produced by:

1. Changing the input-output function of the front-end AGC. There are very few systematic studies exploring the effect on music perception of varying the compression threshold or the compression ratio of the front-end AGC in a CI. Gilbert et al. (2021) explored the effect of varying the compression ratio of the front-end AGC in the Med-El CI, keeping the compression threshold fixed at 48 dB SPL, and found no significant effect on music preferences. However, they varied the compression ratio only over a small range (2.5 to 3.5) and they used samples of music with a single fixed input level of 65 dBA. They did not explore the effect of simultaneously varying the compression threshold and the compression ratio. It seems plausible that a lower compression threshold and a lower compression ratio than currently used would give a better impression of the dynamic changes in a piece of music.
2. Using a multi-channel rather than single-channel front-end AGC system. This would prevent an intense narrowband signal from reducing the level of all frequency components, and it would also, to some extent, reduce the cross-modulation effects described above.
3. For dual time-constant AGC systems, making the slow time constants even slower. However, experimental studies are required to assess whether or not this is beneficial and to determine the optimal compression speed, if there is such a thing.

**Figure 3.**
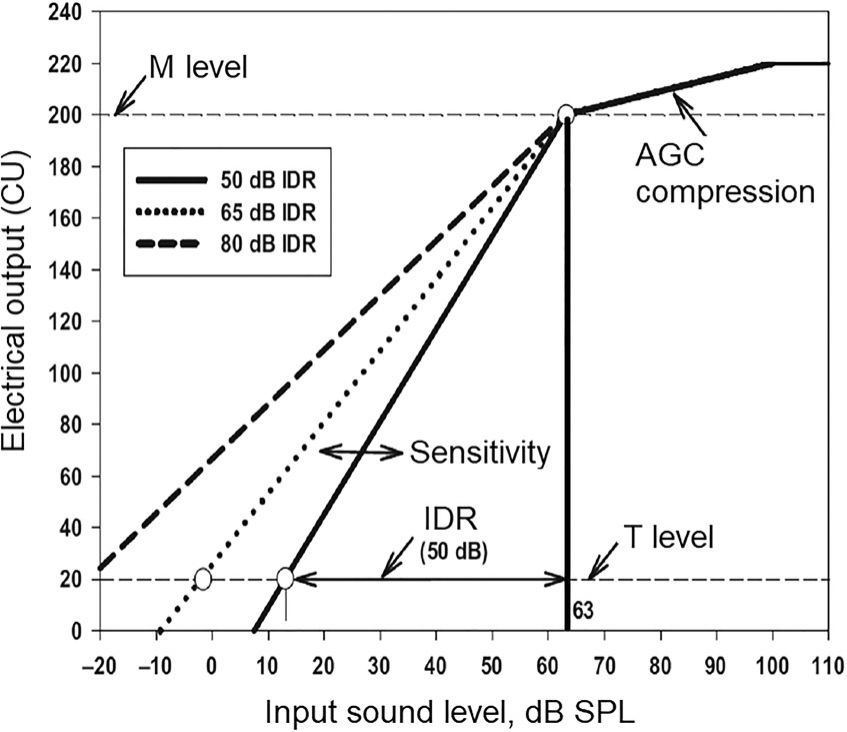
Schematic input-output functions for the advanced bionics CIs for input dynamic ranges (IDRs) of 50, 65, and 80 dB.

As noted above, in addition to the front-end AGC, the CIs of Cochlear Corporation, Med-El, and Advanced Bionics include a stage of instantaneous compression in the transformation from the envelope magnitude in a given channel to the electrical signal applied to the electrode that is driven by that channel. Figure 3 is a schematic illustration of the mapping system used in each channel of an Advanced Bionics CI. The x-axis shows the input sound level. The y-axis shows the magnitude of the electrical signal delivered to the electrode in current units (CU). In this example, the threshold (T) level for that channel is 20 CU and the most comfortable level (M) is 200 CU. The input dynamic range (IDR) is the range of levels that the sound processor codes into electrical stimulation current in the range between the T level and the M level (20 to 200 CU) for each channel. The default IDR is 60 dB, but the IDR can be adjusted via a “sensitivity” control. Figure 3 shows schematic input-output functions for IDRs of 50, 65, and 80 dB. Levels above the top of the IDR are mapped to a very small range of CUs from the M level (200 CU) to an upper limit of about 220 CU (depending on the electrical pulse width and rate). Levels below the bottom of the IDR are mapped to an inaudible current, below the T level. The mapping for input levels above the compression threshold of 63 dB SPL depends on the time-varying gain of the front-end AGC.

In Figure 3, the input-output functions are straight lines when plotted as CU in linear units against effective level in dB. This represents a compressive form of mapping. The shapes of the input output-functions are loosely based on studies of the relationship between electrical signals applied to a single electrode and loudness (Zeng & Shannon, 1995; Zeng et al., 1998). The idea is that loudness fluctuations over time conveyed by changes in the electrical signal applied to a given electrode should resemble the loudness fluctuations that would occur in a normal ear in response to fluctuations in sound level in a local frequency region. In practice, however, the shapes of the functions relating loudness to the electrical signal applied to an electrode can vary from one CI user to another and can vary across electrodes within a CI user. Also, for input signals with levels above the compression threshold (63 dB SPL in this example), which would occur often for live music, much stronger compression is applied. Thus, the mapping process inevitably results in some distortion of the internal representation of the envelope for CI users. Finally, the shapes of the functions mapping envelope magnitude to electrical signal vary across manufacturers (Vaerenberg et al., 2014), and it is not clear what form of mapping gives the most faithful internal representation of the envelope in each channel.

**Figure 4.**
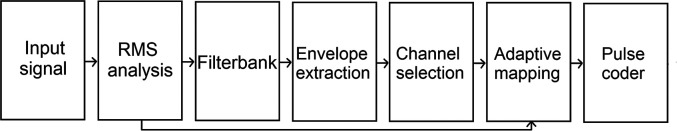
Schematic diagram of the “voice guard” system used in the oticon medical CI.

**Figure 5.**
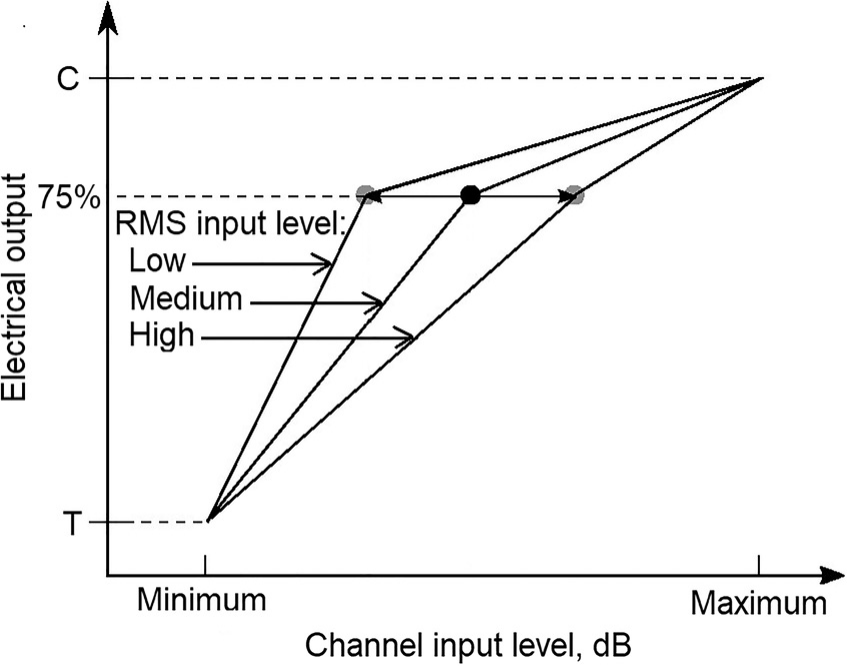
Schematic diagram of the adaptive mapping from the envelope magnitude in a given channel to the electrical output for that channel, as used in the Oticon medical voice guard system.

One manufacturer of CIs, Oticon Medical, uses a different system, which is illustrated in Figure 4, adapted from Langner et al. (2020). This system does not use a front-end AGC. Rather all amplitude compression is applied in the mapping from envelope magnitude in a given channel to the current applied to the corresponding electrode (the electrical pulse width is modulated). This system is called “Voice Guard” since it was designed to convey the envelope fluctuations of speech signals as faithfully as possible. The mapping from envelope amplitude to electrical pulse width is compressive and it is also adaptive. The root-mean-square (RMS) level of the input signal is averaged over the previous 2 s. The estimate of the RMS level is updated every 2 ms. This slowly-changing RMS level estimate is used to control the “knee-point” of the mapping function (similar to the compression threshold) in 3-dB steps, as illustrated in Figure 5. This is done separately for each of four frequency ranges. For a “Low” RMS input level (60 dB SPL), the kneepoint is set to its lowest value (ranging from 52 dB SPL for low centre frequencies to 41 dB SPL for high centre frequencies). For a “High” RMS input level (80 dB SPL), the kneepoint is set to its highest value (ranging from 70 dB SPL for low centre frequencies to 58 dB SPL for high centre frequencies). The system incorporates a “hysteresis” function that prevents the knee-point from fluctuating wildly when the input level is halfway between the values corresponding to two knee-points. The output electrical signal at the kneepoint is always set to 75% of the electrical dynamic range between the threshold (T) and the highest comfortable level (C) for each channel.

As noted above, the Voice Guard system was designed with the intention of improving the perception of speech, especially speech in background noise. As far as I know, it has not been evaluated or optimised for music listening. However, since the mapping in the individual channels is controlled by the slowly-changing RMS level of the broadband signal, the Voice Guard system is likely to suffer from some of the same problems as for the front-end AGCs used in other CI systems. Specifically, if the RMS level of the broadband signal increases, the electrical output decreases for all channels, regardless of whether the increase in RMS level was produced by a narrowband signal (e.g. a strong low-frequency note) or by a broadband signal (e.g. a crescendo in the music). Thus the system will suffer from cross-modulation effects. Also, in the Voice Guard system all of the compression of dynamic range is performed in the instantaneous mapping from channel envelope magnitude to electrical output. The research reviewed above for HAs suggest that for music listening slow compression is preferred over fast compression. Therefore, performing all of the compression via instantaneous mapping is unlikely to be optimal for music listening. Finally, because all of the compression is performed via the mapping, very strong compression has to be used in the mapping. For example, for a high RMS input level of 80 dB SPL, the kneepoint for a channel channel centred close to 2 kHz would be 66 dB. This means that all levels above 66 dB in that channel would be mapped into only 25% of the electrical dynamic range. Potentially, this could reduce the detectability of small envelope fluctuations, although the ability to detect small changes in electric current is usually best towards the upper end of the electrical dynamic range (McKay et al., 2003; Shannon, 1983).

Improvements in the Voice Guard system for music listening might be produced by:

1. Controlling the mapping in individual channels by estimating the running RMS level in different frequency regions and using that to control the mapping in sub-groups of electrodes, rather than by controlling the mapping in all channels by the running RMS level of the broadband signal. For example, the running RMS level at low frequencies would be used to control the mapping for the most apical electrodes.
2. Incorporating a multi-channel slow-acting or dual-time-constant AGC system prior to the instantaneous channel mapping.

### Approaches Based on Loudness Models

Although not implemented in current CI devices, some researchers have explored schemes for CI processing based on loudness models, with aim of restoring the perception of loudness approximately to “normal” for a wide range of input levels and spectra (Francart & McDermott, 2012; McDermott et al., 2003; McKay et al., 2003); for a review, see Wouters et al. (2015). Such schemes are based on loudness models for normal hearing and use the concept of specific loudness, which is the loudness density as a function of centre frequency (Moore, 2014). The aim is to restore the specific loudness pattern to “normal”. The loudness-based schemes have shown promising results for speech in quiet and in noise and for artificial test signals, but they have not, to my knowledge, been comprehensively evaluated using music.

## Issues Associated with Binaural CIs

Many people with bilateral severe or profound hearing loss are fitted with bilateral CIs. The use of bilateral CIs can improve the ability to understand speech in the presence of background sounds and can also improve the ability to localise sounds (Culling et al., 2012; Dunn et al., 2008; Litovsky et al., 2009). The improvement in localisation appears to depend largely on the use of interaural level difference (ILD) cues (Litovsky et al., 2009; Seeber & Fastl, 2008). Currently, the slowly-adapting AGC systems that are used in CIs operate independently at the two ears. The use of independent AGC at the two ears distorts interaural level difference (ILD) cues for sound localisation (Wiggins & Seeber, 2011), especially when the head is moved (Archer-Boyd & Carlyon, 2019; Archer-Boyd & Carlyon, 2021). The magnitude of the effect of head movement varies in a complex way with the attack and release time of the AGC system and with the speed and type of movement of the head of the CI user. This disrupts ILD cues for sound localisation, especially dynamic cues.

In principle, the distortion of dynamic ILD cues can be reduced by linking the AGC across ears, as is done in some HAs. When the AGC is linked, the same gain-control signal is applied to the signal at each ear, preserving ILD cues. The evidence for benefits of linked multi-channel AGC in HAs is mixed, and when slow-acting AGC is used the benefits appear to be minimal (Moore et al., 2016; Wiggins & Seeber, 2013). One study has reported benefits of synchronisation of the front-end AGC in experimental versions of bilaterally fitted Advanced Bionics CIs for the localisation of both static and moving sound sources (Dwyer et al., 2021). However, in that study the participants did not move their heads. Also, it is not clear whether the benefits found were a result of better preservation of ILD cues because of the synchronisation, or whether they resulted from the fact that synchronisation of AGC systems generally results in less overall compression and more slowly changing compression. Finally, it should be noted that linking of AGC systems distorts the trajectory of the changes in level at each ear as the user moves their head (Archer-Boyd & Carlyon, 2021); in other words, monaural envelope cues are distorted. Clearly, more research is needed into the potential benefits of linking the AGC systems in bilaterally fitted CIs, especially in the context of music perception.

## Conclusions

This paper has reviewed the AGC systems used in HAs and has considered how some of the lessons learned from studies of the AGC systems in HAs might be applied to improving music listening for users of CIs. The following recommendations are made:

1. A CI should be set up with a dedicated music program and any adaptive directional processing and noise reduction should be disabled for that program.
2. The dynamic range of the input stage of CIs should be increased, either by the use of ADCs with more resolution than 16 bits or via the use of a controllable gain applied to the microphone signal(s) before analogue-to-digital conversion.
3. There is a need for systematic studies exploring the effect on music perception of varying the compression threshold and the compression ratio of the front-end AGC used in the CIs of several manufacturers. It seems plausible that a lower compression threshold and a lower compression ratio than currently used would give a better impression of the dynamic changes in a piece of music, but this remains to be assessed, using both recorded and live music.
4. Experimental studies comparing multi-channel and single-channel front-end AGC systems for music listening should be conducted. In principle the use of multi-channel AGC would prevent an intense narrowband signal from reducing the effective level of all frequency components, and it would also, to some extent, reduce cross-modulation effects between different sound sources.
5. For dual time-constant AGC systems, the effect of making the slow time constants even slower should be explored.
6. For the system in which the running RMS level of input signal is used to control the kneepoint of the mapping function, the potential benefits should be explored of controlling the mapping in individual channels by estimating the running RMS level in different frequency regions and using that to control the mapping in sub-groups of electrodes, rather than by controlling the mapping in all channels by the running RMS level of the broadband signal. As for multi-channel AGC, this should prevent an intense narrowband signal from reducing the effective level of all frequency components, and it should also, to some extent, reduce cross-modulation effects between different sound sources.
7. For the the system in which the running RMS level of input signal is used to control the kneepoint of the mapping function, the potential benefits should be explored of incorporating a multi-channel slow-acting or dual-time-constant AGC system prior to the instantaneous channel mapping. This would reduce the amount of instantaneous compression required for the mapping in each channel, helping to preserve short-term intensity contrasts and amplitude-modulation patterns.
8. Research is needed into the potential benefits of linking the AGC systems in bilaterally fitted CIs, especially in the context of music perception.
9. Users of bilateral CIs should be cautioned that rapid head movements might affect the apparent positions of sounds in space. They should be advised to move their heads only slowly when listening to music.
